# Topical Imiquimod for the Treatment of Relapsed Cutaneous Langerhans Cell Histiocytosis after Chemotherapy in an Elderly Patient

**DOI:** 10.1155/2018/1680871

**Published:** 2018-01-03

**Authors:** Shinsaku Imashuku, Miyako Kobayashi, Yoichi Nishii, Keisuke Nishimura

**Affiliations:** ^1^Department of Laboratory Medicine, Uji-Tokushukai Medical Center, Uji 611-0042, Japan; ^2^Department of Internal Medicine, Uji-Tokushukai Medical Center, Uji 611-0042, Japan; ^3^Division of Plastic Surgery, Uji-Tokushukai Medical Center, Uji 611-0042, Japan; ^4^Department of Pathology, Uji-Tokushukai Medical Center, Uji 611-0042, Japan

## Abstract

Diagnosis and treatment of Langerhans cell histiocytosis (LCH) in elderly patients are often difficult. We report here a 61-year-old female suffering from a refractory axillary ulcer for nearly a year, whose biopsy revealed LCH. It was also noted that the patient had other cutaneous papulovesicular eruptions of LCH as well as central diabetes insipidus. The patient was first successfully treated with multiagent chemotherapy (cytosine arabinoside/vinblastine/prednisolone). DDAVP also well controlled diabetes insipidus; however, the axillary ulcer and cutaneous LCH relapsed. Thereafter, we found topical imiquimod to be effective in the treatment of relapsed cutaneous LCH lesions.

## 1. Introduction

Langerhans cell histiocytosis (LCH) is a rare disease characterized by granulomatous lesions consisting of clonal CD1a+/CD207+/S100+ immature dendritic cells and various inflammatory cells. Currently, LCH is defined as inflammatory myeloid neoplasia [1]. Approximately two-thirds of LCH cases occur in pediatric patients, while the remaining one-third occur in adult patients. In an analysis of 275 adults with LCH, involvement of the lungs was the highest (58.4%), followed by bone (57.3%), skin (36.9%), and central diabetes insipidus (29.6%) [2]. However, LCH in adults is often misdiagnosed because of its rarity, particularly cutaneous lesions, which affect the scalp, neck, axilla, groin, and trunk with various forms from papules to vesicles; thus, if not biopsied, cutaneous LCH is overlooked as nonspecific eruptions. In terms of treatment of LCH, multiagent chemotherapy is employed for systemic multifocal lesions [3, 4]. On the other hand, for isolated cutaneous LCH, oral or topical steroids are considered as first-line treatment [5]; however, the appropriate therapy for refractory cutaneous LCH cases remains controversial. To date, various therapies such as topical nitrogen mustard [6] or thalidomide [7] and systemic low-dose methotrexate [8] or interferon- (IFN-) alpha [9] were reported. In addition, although numbers are limited, the effectiveness of topical imiquimod treatment was described [10–13]. Here, we report on an elderly patient whose relapsed, postchemotherapy cutaneous LCH lesions were successfully treated with topical imiquimod.

## 2. Case Report

**Figure 1:**
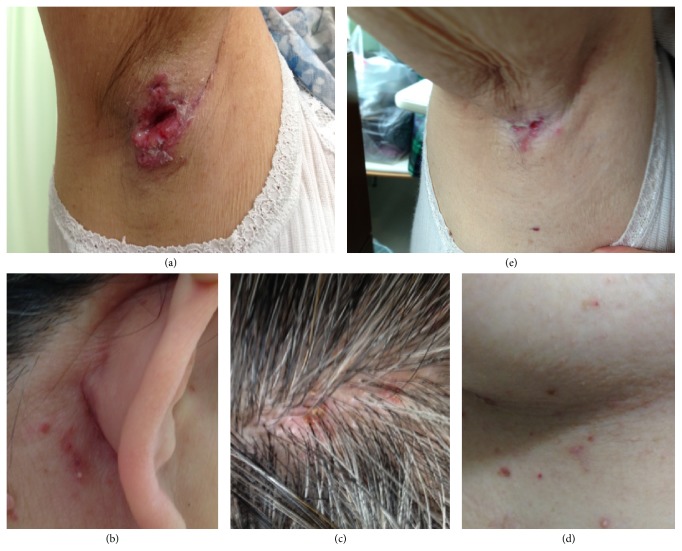
Photos of pretreatment right-axillary ulcer (a); cutaneous eruptions of LCH at the retroauricular area (b), scalp (c), and under the breast (d); posttreatment (after 4.5 months of imiquimod) status at the right axilla (e).

**Figure 2:**
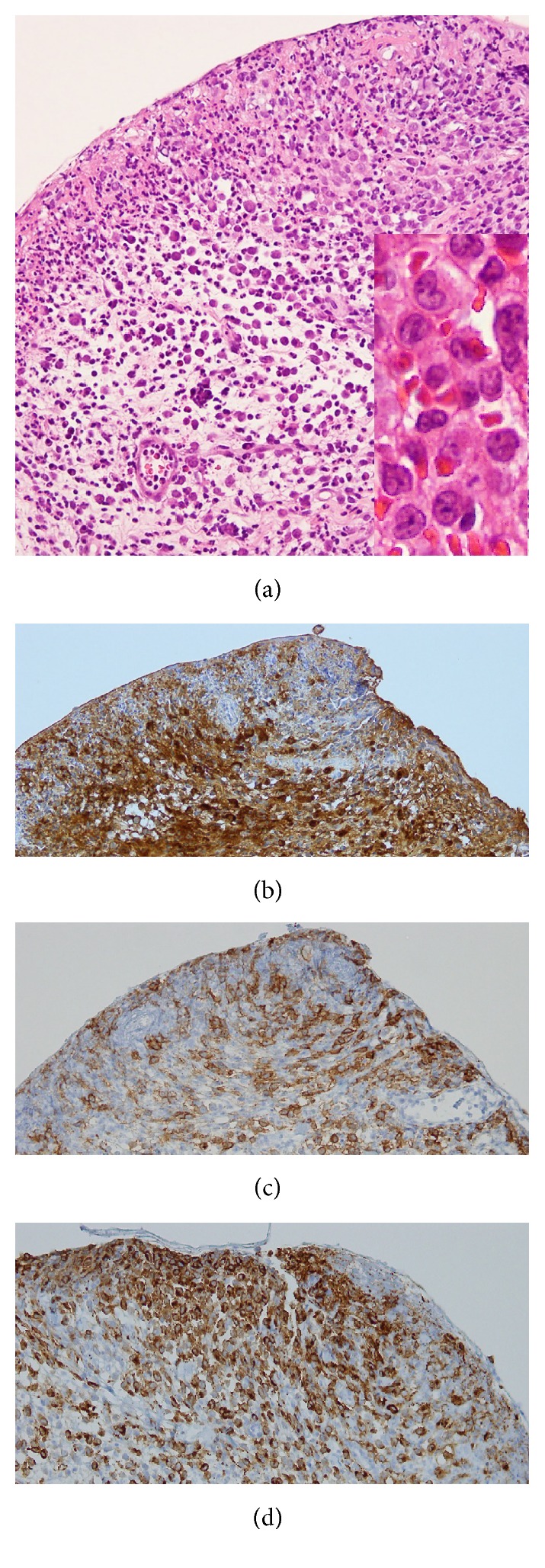
Pathology of the biopsied pretreatment axillary ulcer. H&E stain (a; original magnification ×200 with magnified photo showing LCH cells with folded coffee bean-like nucleus, ×400) and immunostaining of S100-positive (b), CD1a-positive (c), and CD207-positive (d) LCH cells (original magnification: ×200).

**Figure 3:**
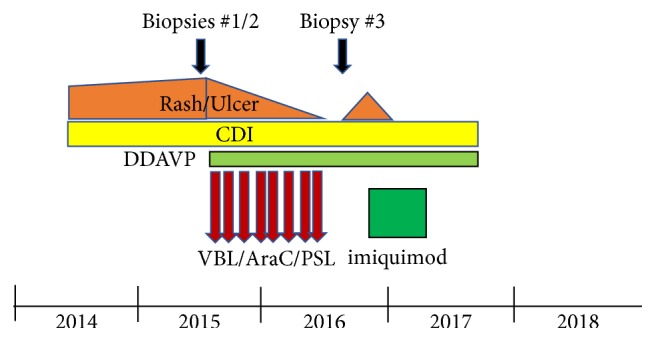
Clinical course and treatment. Biopsies #1/2 were done on the axillary ulcer and the retroauricular cutaneous lesions and biopsy #3 was done on the relapsed retroauricular cutaneous lesion. Diagnosis of LCH was made after biopsy #1. Pathological findings of #2 and #3 were almost identical to those of #1 shown in Figure 2. Details of the chemotherapy (VBL/AraC/PSL) and imiquimod regimens are described in the text. Symptoms of central diabetes insipidus were controlled by DDAVP.

The case described here is a 61-year-old Japanese female who had been treated for diabetes mellitus and a refractory large ulcer (2.0 cm × 2.6 cm) at her right axilla (Figure 1(a)) for nearly a year. Eventually, the ulcer was biopsied, revealing a typical LCH pathology, with dermal infiltrate of morphologically characteristic Langerhans cells extending into the epidermis, which were positive for S100, CD1a, and CD207, with other inflammatory cells (Figure 2). Prior to the diagnosis, cutaneous eruptions, such as erythematous papules/vesicles at retroauricular regions, crusted papules at the scalp, and reddish-brown papules at the lower chest under the breasts, were present (Figures 1(b), 1(c), and 1(d)). These cutaneous lesions remained undiagnosed until a biopsy of the retroauricular papule was performed which also revealed an LCH pathology. It was thought that one of such cutaneous eruptions caused a deep ulcer in the axillary. Thereafter, we examined whether the patient had systemic LCH lesions in the lungs, bones, and other organs. None was found except for abnormal brain MRI findings showing a thickened pituitary stalk and absent high signal at the posterior lobe of pituitary on T1WI (figure not shown). Based on her symptoms of polyuria/polydipsia, she was diagnosed with LCH-related central diabetes insipidus. Serum levels of antidiuretic hormone were undetectable (<0.8; reference value: >4.2 pg/mL). The reason for the delayed diagnosis of central diabetes insipidus was because her physician had been so concerned about treating axillary ulcer and her occasional complaints of polyuria/polydipsia were thought to be due to diabetes mellitus. After the diagnosis of LCH, we chose to treat this patient systematically, because the axillary ulcer was so deep and enlarged (see Figure 1(a)) along with the presence of CNS lesion. The patient underwent treatment with DDAVP for central diabetes and systemic chemotherapy consisting of (I) vinblastine (VBL; 8 mg/day, intravenous infusion) and prednisolone (PSL; 30 mg/day, intravenous infusion) on Day 1 and (II) cytosine arabinoside (AraC; 200 mg/day, intravenous infusion) and PSL (30 mg/day, intravenous infusion) on Day 2 every 4 weeks. After 8 cycles of chemotherapy, the axillary ulcer healed and cutaneous lesions disappeared; however, the thickened pituitary stalk in the CNS was unchanged. Three months later, the axillary ulcer relapsed and cutaneous eruptions reappeared, and diagnosis of LCH was again confirmed by a biopsy (Figure 3). Central diabetes insipidus did not exacerbate and no increase of DDAVP dose was required. This time, considering the adverse effects of the previous systemic chemotherapy (glucose intolerance in the presence of diabetes mellitus and progressive dementia), we chose to employ topical imiquimod to treat the relapsed axillary ulcer and cutaneous LCH lesions, in addition to continuation of DDAVP for central diabetes insipidus. In this case, imiquimod (5%) cream was applied 5 days a week to the right axilla, scalp, and retroauricular areas and lower chest lesions, according to the instructions of the manufacturer. Following treatment for 4 months, the relapsed axillary ulcer as well as other cutaneous lesions improved significantly (Figure 1(e)). During the total 6.5 months of imiquimod treatment, no adverse effects such as fever or cutaneous redness, sore, and exfoliation were noted. At the time of writing this paper, more than 8 months after stopping the imiquimod treatment, no further relapse of the axilla and other cutaneous lesions was noted. We assessed the therapeutic results as a clinical remission, since no biopsy was performed to confirm the complete loss of LCH cells, as summarized in Table 1.

## 3. Discussion

Imiquimod, a cytokine inducer and a modifier of the innate immune response [14], is approved in Japan for the treatment of genital warts and actinic keratosis. Imiquimod is believed to function as a potent stimulator of T-helper-1 cytokines causing local release of IFN-alfa, tumor necrosis factor-alpha, interleukin-1, interleukin-6, and others [15]. In LCH, it was postulated that topical imiquimod might be effective through IFN-alpha, but sparing patients the systemic side effects of IFN treatment [10]. Although imiquimod cream was not approved as an agent for LCH, we employed it in this case because its efficacy was reported in the past in the treatment of cutaneous LCH [10–13]. Including our case, topical imiquimod was employed for the duration of 1 month to 6.5 months in two pediatric and three adult patients, in whom no significant side effects were reported, and all showed complete histological or clinical remission, although two showed a relapse after 6 months (see Table 1).

This case illustrates the difficulty of diagnosing LCH in elderly patients and the usefulness of topical imiquimod for relapsed cutaneous LCH after chemotherapy. The good outcome in this case may have been the result of the additive effect of the initial systemic chemotherapy and later imiquimod treatment; however, it is emphasized that the rapid response of relapsed cutaneous LCH to topical imiquimod was obtained after short-term administration. Although the patient's condition has been stable, considering previous reports, she may still have a risk of relapse in the future after imiquimod treatment. In view of the risk of recurrence and subsequent malignancy in adult LCH [16], it is recommended that this case requires further long-term follow-up. Finally, we think that it is worth testing more this topical imiquimod in the future in the treatment of cutaneous LCH, particularly in elderly patients who are not tolerable to chemotherapy, considering its easy availability and effectiveness without significant side effects.

## Figures and Tables

**Table 1:** Report of topical imiquimod trials for cutaneous LCH.

References	Case (age/gender)	Disease	Previous Rx		Topical imiquimod; duration (response)	Follow-up/outcome after finishing imiquimod
			Systemic chemotherapy	Topical Rx for skin LCH		
Dodd and Hook [13]	16 mo/F	Isolated skin LCH alone	None	Corticosteroids/tacrolimus.	5 months (CR)	>2 yrs No relapse

Aubert-Wastiaux et al. [12]	4 yr/M	Simultaneous skin LCH with T-ALL	For T-ALL	None	1 month (CHR)	Aggressive LCH Died in <2 months

O'Kane et al. [11]	53 yr/F	Breast carcinoma, followed by isolated skin LCH	For breast carcinoma	None	6 weeks (CHR)	Relapse after 6 months and then repeat imiquimod CR for 12 months

Taverna et al. [10]	74 yr/F	Isolated skin LCH alone	None	Ketoconazole/hydrocortisone	2 months (CR)	Relapse after 6 months and then repeat imiquimod

Current	61 yr/F	Skin LCH/CDI	For LCH ulcer	None	6.5 months (CR)	>8 months No relapse

LCH: Langerhans cell histiocytosis; ALL: acute lymphocytic leukemia; CDI: central diabetes insipidus; Rx: treatment; CR: clinical remission (not confirmed by biopsy after treatment); CHR: complete histological remission (confirmed by biopsy after treatment).

## References

[1] Berres M. L., Merad M., Allen C. E. (2015). Progress in understanding the pathogenesis of Langerhans cell histiocytosis: back to Histiocytosis X?. *British Journal of Haematology*.

[2] Aricò M., Girschikofsky M., Généreau T. (2003). Langerhans cell histiocytosis in adults. Report from the International Registry of the Histiocyte Society. *European Journal of Cancer*.

[3] Minkov M., Grois N., Heitger A., Potschger U., Westermeier T., Gadner H. (2000). Treatment of multisystem Langerhans cell histiocytosis. Results of the DAL-HX 83 and DAL-HX 90 studies. *Klinische Pädiatrie*.

[4] Morimoto A., Shioda Y., Imamura T. (2016). Intensified and prolonged therapy comprising cytarabine, vincristine and prednisolone improves outcome in patients with multisystem Langerhans cell histiocytosis: results of the Japan Langerhans Cell Histiocytosis Study Group-02 Protocol Study. *International Journal of Hematology*.

[5] Park L., Schiltz C., Korman N. (2012). Langerhans cell histiocytosis. *Journal of Cutaneous Medicine and Surgery*.

[6] Lindahl L. M., Fenger-Grøn M., Iversen L. (2012). Topical nitrogen mustard therapy in patients with Langerhans cell histiocytosis. *British Journal of Dermatology*.

[7] Sander C. S., Kaatz M., Elsner P. (2004). Successful treatment of cutaneous langerhans cell histiocytosis with thalidomide. *Dermatology*.

[8] Steen A. E., Steen K. H., Bauer R., Bieber T. (2001). Successful treatment of cutaneous Langerhans cell histiocytosis with low-dose methotrexate. *British Journal of Dermatology*.

[9] Chang S. E., Koh G. J., Choi J. H. (2002). Widespread skin-limited adult Langerhans cell histiocytosis: Long-term follow-up with good response to interferon alpha. *Clinical and Experimental Dermatology*.

[10] Taverna J. A., Stefanato C. M., Wax F. D., Demierre M.-F. (2006). Adult cutaneous Langerhans cell histiocytosis responsive to topical imiquimod. *Journal of the American Academy of Dermatology*.

[11] O'Kane D., Jenkinson H., Carson J. (2009). Langerhans cell histiocytosis associated with breast carcinoma successfully treated with topical imiquimod. *Clinical and Experimental Dermatology*.

[12] Aubert-Wastiaux H., Barbarot S., Mechinaud F. (2011). Childhood Langerhans cell histiocytosis associated with T cell acute lymphoblastic leukemia. *European Journal of Dermatology*.

[13] Dodd E., Hook K. (2016). Topical Imiquimod for the Treatment of Childhood Cutaneous Langerhans Cell Histiocytosis. *Pediatric Dermatology*.

[14] Vidal D. (2006). Topical imiquimod: Mechanism of action and clinical applications. *Mini-Reviews in Medicinal Chemistry*.

[15] Imbertson L. M., Beaurline J. M., Couture A. M. (1998). Cytokine induction in hairless mouse and rat skin after topical application of the immune response modifiers imiquimod and S-28463. *Journal of Investigative Dermatology*.

[16] Edelbroek J. R., Vermeer M. H., Jansen P. M. (2012). Langerhans cell histiocytosis first presenting in the skin in adults: Frequent association with a second haematological malignancy. *British Journal of Dermatology*.

